# Doctoral training in Uganda: evaluation of mentoring best practices at Makerere university college of health sciences

**DOI:** 10.1186/1472-6920-14-9

**Published:** 2014-01-13

**Authors:** Damalie Nakanjako, Achilles Katamba, Dan K Kaye, Elialilia Okello, Moses R Kamya, Nelson Sewankambo, Harriet Mayanja-Kizza

**Affiliations:** 1 Department of Internal Medicine, Makerere University College of Health Sciences, Kampala, Uganda; 2 Department of Obstetrics and Gynaecology, Makerere University College of Health Sciences, Kampala, Uganda; 3 Department of Psychiatry, Makerere University College of Health Sciences, Kampala, Uganda

**Keywords:** Mentorship, Doctoral training, Supervision, Capacity building, Health care, Low and middle income countries, Uganda

## Abstract

**Background:**

Good mentoring is a key variable for determining success in completing a doctoral program. We identified prevailing mentoring practices among doctoral students and their mentors, identified common challenges facing doctoral training, and proposed some solutions to enhance the quality of the doctoral training experience for both candidates and mentors at Makerere University College of Health Sciences (MakCHS).

**Methods:**

This cross-sectional qualitative evaluation was part of the monitoring and evaluation program for doctoral training. All doctoral students and their mentors were invited for a half-day workshop through the MakCHS mailing list. Prevailing doctoral supervision and mentoring guidelines were summarised in a one-hour presentation. Participants were split into two homogenous students’ (mentees’) and mentors’ groups to discuss specific issues using a focus group discussion (FGD) guide, that highlighted four main themes in regard to the doctoral training experience; what was going well, what was not going well, proposed solutions to current challenges and perceived high priority areas for improvement. The two groups came together again and the note-takers from each group presented their data and discussions were recorded by a note-taker.

**Results:**

Twelve out of 36 invited mentors (33%) and 22 out of 40 invited mentees (55%) attended the workshop. Mentors and mentees noted increasing numbers of doctoral students and mentors, which provided opportunities for peer mentorship. Delays in procurement and research regulatory processes subsequently delayed students’ projects. Similarly, mentees mentioned challenges of limited; 1) infrastructure and mentors to support basic science research projects, 2) physical office space for doctoral students and their mentors, 3) skills in budgeting and finance management and 4) communication skills including conflict resolution. As solutions, the team proposed skills’ training, induction courses for doctoral students-mentor teams, and a Frequently Asked Questions’ document, to better inform mentors’, mentees’ expectations and experiences.

**Conclusion:**

Systemic and infrastructural limitations affect the quality of the doctoral training experience at MaKCHS. Clinical and biomedical research infrastructure, in addition to training in research regulatory processes, procurement and finance management, communication skills and information technology, were highlighted as high priority areas for strategic interventions to improve mentoring within doctoral training of clinician scientists.

## Background

Over the last decade, the number of health care doctoral students at Makerere University College of Health Sciences (MakCHS) has increased by over 10 fold with the increasing funding opportunities for capacity building for health care and health leadership [1,2]. Funding for doctoral training had been realised through collaborative capacity building programs including among others; Medical Education Partnership Initiative (MEPI)–Medical Education for All Ugandans (MESAU), Training Health Researchers into Vocational Excellence (THRIVE), Malaria Capacity Development Consortium (MCDC), Millenium Science Initiative (MSI), Netherlands University Foundation for International Cooperation (NUFFIC) and Swedish International Development Agency (SIDA). Each of these collaborative doctoral programs provides unique features that could be utilised to promote mentoring best practices for doctoral students at MakCHS. For example, a majority of the programs combine local and international supervision, with mentors from partner institutions, thereby providing a global perspective to the local doctoral training program. In addition, it is likely that the programs face some similar challenges since they all involve students at MakCHS and other medical schools in Uganda.

Mentoring best practices are critical to the sustainability of training clinicians, academicians, educators and researchers to understand and take up the critical gaps in global health leadership in developing countries [3,4]. Mentoring for doctoral students is the alliance between the doctoral student and his/her major dissertation advisors, supervisors and/or mentors [5]. During doctoral training, the mentoring process includes choosing the mentor, formalizing of the mentoring alliance, discussion of the mentor/mentee roles, and the evaluation of the mentoring process [5]. A good mentoring process is a key variable for determining success in completing a doctoral program. Given the high investment in doctoral training, the current global climate of diminishing resources and the great need for building local capacity for higher education, understanding and examining the challenges to students’ ability to complete their doctoral degree requirements in a timely manner remains critical [6].

There are general guidelines for doctoral training at MakCHS that outline the various processes ranging from registration to completion as well as requirements for doctoral students and supervisors. However, implementation of the doctoral guidelines faces various challenges that may delay completion of doctoral training. Considering the limited pool of mentors at MAKCHS, it remains critical to equip doctoral supervisors/advisors with skills to develop mutually beneficial mentoring relationships with their doctoral trainees [4]. We identified prevailing mentoring practices among the doctoral candidates and their mentors, identified the common challenges facing doctoral training, and proposed solutions to enhance the quality of the doctoral training experience for both the candidates and mentors at Makerere University and affiliated institutions. Strategies from this model could be scaled up in the region through the collaborative programs that involve students and mentors from several Universities within the country, the region and overseas.

## Methods

### Study setting and participants

This evaluation was conducted as part of the monitoring and evaluation program for doctoral training at MakCHS. A team of four faculty members attended a trainer of trainer (TOT) course in doctoral supervision as part of the MCDC capacity building program in Africa. Upon their return from Dakar, Senegal, this committee planned a mentoring workshop for all doctoral students and doctoral supervisors/advisors within the various doctoral programs at MakCHS including; MEPI-MESAU, THRIVE, MSI and SIDA. The main aim of the workshop was to identify the best practices that should be maintained, identify the main challenges, and facilitate the mentors’ and students’ teams to propose solutions as well as identify the next high priority areas to improve the quality of the doctoral training experience at MakCHS.

### Procedures

In a cross-sectional qualitative evaluation, all doctoral students and their mentors were invited to a half-day workshop on ‘PhD supervision best practices’ through the college mailing list. The first hour of the workshop was used to generate a common understanding of the goals of the workshop, summarise the prevailing doctoral supervision and mentoring guidelines at MakCHS, and put subsequent discussions within the institutional operational context. The doctoral students and supervisors were then split into two homogenous groups to discuss specific issues for 60 minutes, as directed by the focus group discussion (FGD) guide. The FGD guide covered four main themes in regard to the doctoral training experience; what was going well, what was not going well, proposed solutions for the current challenges and what were considered as the highest priority areas to improve the doctoral training experience? These themes were derived from the college doctoral training committee meetings on monitoring and evaluation to inform subsequent steps in doctoral program enhancement. The data was recorded by a note-taker in each of the groups. The two groups came together again and the note-takers from each group presented their data for a 30-minute discussion by the combined team of mentors and mentees. The combined group discussion was also recorded by a note-taker. Data analysis was conducted using the principles of thematic analysis [7-9]. Data was analysed manually according to the pre-determined themes; summarised in tables and quotes reported verbatim. This work was done in compliance with the Helsinki Declaration (http://www.wma.net/en/30publications/10policies/b3/index.html), with approval sought from the Makerere University College of health Sciences Institutional Review Board.

## Results

Overall, 12 out of 36 invited mentors (33%) and 22 out of 40 invited mentees (55%) attended the workshop, representing at least eight disciplines within the college of health sciences; Nursing, Basic sciences, Internal Medicine, Psychiatry, Paediatrics, Surgery, Obstetrics & Gynaecology and Public Health (Table 1). The students had between 1–7 years’ experience in the PhD programme and the mentors had mentoring experience of 1–10 doctoral students.

**Table 1:** Participants of the doctoral mentorship workshop at Makerere University College of Health Sciences

	**Mentors**	**Doctoral students**
	**N = 12**	**N = 22**
**Gender**		
Female n (%)	5(42)	12(54)
**Disciplines represented¶**		
Internal medicine	1	5
Psychiatry	3	
Paediatrics	2	
Surgery		2
Obstetrics & Gynaecology	3	5
Basic sciences	1	3
Public Health		2
Epidemiology	1	4
Nursing	1	1
Others*		

*Three research administrators and one information technology expert.

¶Representation of the mentors and doctoral students by discipline.

### What was going well?

Doctoral students noted the increasing numbers of doctoral students which provides them several opportunities for peer support through meetings such as the ‘PhD Forum’ where they meet to discuss their projects as well as challenges. ‘*The PhD forum allows us to support each other to deal with difficult situations’* said one doctoral student. They also noted the gradually increasing number of committed mentors for the doctoral students (Table 2).

**Table 2:** Doctoral students’ discussions on mentoring through doctoral training at Makerere University College of Health Sciences

**Thematic questions**	**Responses**
**What is going well?**	● Peer support received from the increasing number of doctoral candidates in the various disciplines.
	● Available guidelines for doctoral supervision and mentoring.
	● Increasing numbers of supervisors and their commitment to mentor doctoral candidates.
	● PhD forum where students have opportunities to present their research projects.
**What is not going well?**	
**Physical space**	● Limited physical space for doctoral students in their respective departments.
	● Limited physical space for the mentors in their respective departments.
**Procurement**	● Unexplained delays in the procurement processes which, in many cases, delay the students’ progress.
**Registration processes**	● Lengthy registration processes including delays in appointing doctoral supervisors.
**Biomedical sciences**	● Limited number of mentors with the skills to supervise and mentor doctoral students.
	● Limited laboratory infrastructure for biomedical research so that most students have to ship samples abroad.
**Proposed solutions**	
**Infrastructure development**	● Improvement of laboratory infrastructure to support doctoral students’ projects locally.
	● Infrastructure development to create office space for doctoral students during their doctoral period.
	● Create office space for post-doctoral and faculty involved in mentoring doctoral students and junior faculty.
**Procurement processes**	● Improve the procurement systems to meet the needs of doctoral students and other scientists in biomedical research at Makerere University.
**Registration processes**	● Induction courses for doctoral students and their mentors, including a mandatory course on PhD supervision.
	● A catalogue of frequently asked questions (FAQs) for doctoral Students and mentors should be compiled and uploaded on the institutional website.
	● Regular PhD forum meetings and seminars.
**Communication**	● Communication skills’ training for both mentors and mentees including skills in conflict resolution.
**Others**	● Facilitate more seminars between mentors and doctoral students for inspiration and encouragement through the doctoral training experience.

Similarly, the mentors noted the increasing number of doctoral students as well as the increasing funding opportunities for doctoral training at MakCHS, that enabled more students to complete doctoral training within 3–5 years since their research is funded. ‘*With available funding for research projects, our students are able to complete doctoral training within 3–5 years*’ said one mentor. In addition, the mentors mentioned that mentoring of doctoral students contributes to the faculty’s score for promotion (Table 3).

‘*I am encouraged to supervise and mentor doctoral students as it contributes to my promotion and professional development’*, said one mentor.

**Table 3:** Mentors’ discussions of the current status and proposed solutions to improve mentoring through doctoral training at Makerere University College of Health Sciences

**Thematic questions**	**Responses**
**What is going well?**	
	There are more numbers of doctoral students, which has increased the opportunities for peer support.
	Supervisors of the doctoral students are more tuned to supervise and mentor students better than before.
	Funding opportunities for doctoral research have increased.
	More students complete doctoral training within 3–5 years because of the available opportunities to fund their research projects.
	Supervision of doctoral students is one of the recognised parameters for promotion.
**What is not going well?**	
	Delays in the procurement systems cause unnecessary delays in students’ research progress.
	There are limited systems for international procurement of laboratory-based research supplies which causes huge delays for basic science research projects.
	Unexplained delays by the national drug authority (NDA) for research projects including investigational drugs.
	Limited human resource for health care when clinicians are pursuing doctoral training activities.
	Limited communication between doctoral students and their mentors.
**Proposed solutions**	
**Procurement delays**	● Conduct workshops between the scientists [doctoral students and mentors] and the institutional procurement team.
	● Propose and design institutional procurement systems with provisions for international procurement of laboratory-specific equipment and consumables.
**Limited communication between mentors and mentees**	● Strategic approach to implement the institutional doctoral supervision and mentoring guidelines among doctoral students and mentors.
	● Communication skills’ training including skills in conflict resolution.
	● Induction courses for doctoral students and mentors.
	● Utilisation of the College of Health Sciences’ research support centre.
**Others**	● Clinical departments need to relieve doctoral students of some clinical duties to allow some protected time for academic research training although the students might still participate in selected teaching activities due to limited teaching staff.
	● Facilitate more seminars to increase networking between doctoral students and mentors.

### What was not going well?

Doctoral students mentioned the challenge of limited physical space for the doctoral students and some of the mentors in their respective departments. One doctoral student said, ‘*Some of our mentors still occupy the space for doctoral students because they do not have office space in their respective departments*’. Students identified unnecessary delays in the procurement processes as a sticking point in the training process. *‘There are unexplained delays in the procurement processes which, in many cases, delay our progress’* said one doctoral student. Also mentioned, was the quite lengthy registration processes, including the appointment of doctoral supervisors which also kept the students behind schedule. Lastly, the students involved in biomedical research noted the limited number of mentors with the skills to supervise and mentor doctoral students in this field as well as the limited laboratory infrastructure. One doctoral student said, ‘*Most of us have to ship our samples abroad because we do not have the required facilities to do our assays locally’* (Table 2).

Similar to the students, the mentors re-echoed the unnecessary delays in the procurement systems, more so for laboratory-based research supplies which caused huge delays for the basic science research projects. In addition, mentors highlighted the challenge of unexplained delays by the National Drug Authority (NDA) which affected most studies involving investigational drugs. Furthermore, the mentors from the clinical disciplines noted the challenge of limited human resources for health care delivery when clinicians are undertaking their doctoral training. Lastly, it was noted that there was limited communication between the doctoral students and their mentors which in some cases delayed the students’ progress if he/she had unresolved challenges (Table 3).

### Proposed solutions to improve the mentoring experience during doctoral training

#### Infrastructure development

Doctoral students highlighted the need for targeted investment towards the improvement of laboratory infrastructure to support doctoral students’ projects locally. In addition, the students proposed physical expansion to create office space for the increasing numbers of doctoral students as well as their mentors most of which are junior and senior faculty members*. ‘We do not have offices because previous doctoral students still occupy the space and have nowhere to move to’* said one doctoral student.

#### Procurement processes

Doctoral students proposed improvement of procurement systems to meet the needs of students and other scientists in biomedical research at Makerere University (Table 2). Similarly, the mentors proposed workshops between the procurement team and the scientists so that both can appreciate and put systems in place to meet each others’ needs in a timely fashion (Table 3). *‘We need to let the procurement team know that the system does not meet our needs*’, said one mentor.

#### Registration processes

Doctoral students proposed regular induction courses for all doctoral students and their mentors at MakCHS, including mandatory training in PhD supervision and mentorship. In addition, they felt that a catalogue of frequently asked questions (FAQs) for doctoral students should be compiled and provided on the doctoral students’ website since many of the FAQs might cut across the different disciplines. Doctoral students also encouraged more regular seminars for doctoral students to share their challenges and get supported with solutions.

#### Communication

Both students and mentors reported the prevailing limited communication between doctoral students and their mentors, and highlighted the need for training in communication skills including conflict resolution*. ‘I need to know what to do if my supervisors disagree and I fail to move on with my project*’ said one doctoral student.

### High priority areas for mentoring workshops as identified by doctoral students and mentors

#### Workshops on procurement processes

The mentors noted that it was critical to facilitate workshops with the procurement team not only to orientate the scientists on the relevant procurement processes but also to orientate the procurement team on the specific needs of the scientists at the college of health sciences in order to foster mutual benefit.

#### Training in finance management and budgeting

Identified as high-priority, was the need to equip doctoral students with skills to design, implement and manage their budgets in good alignment with their research projects.

#### Research regulatory procedures

The team emphasised the need for scheduled meetings with research regulatory bodies to share feedback from the researchers and vise-versa. A case in point was the National Drug Authority (NDA), which was sighted as a common cause of unexplained delays in approval of research involving investigational drugs. The mentors proposed follow-up meetings to engage NDA management and come up with more appreciable timelines as well as strategies to support the two bodies to meet the desired timelines and avoid unnecessary delays to the research work. Besides NDA, doctoral students needed ongoing training in human subjects’ research as well as regular updates on the research regulatory processes by the Institutional Review Boards (IRBs) and Uganda National Council for Science and Technology (UNCST).

#### Communication skills

With communication challenges highlighted by both the doctoral students’ and mentors’ groups, training in communication skills was noted as an important skill to keep doctoral students, mentors, collaborators and the entire research team in harmony in order to achieve the common goals of their research teams (Table 4). ‘*We need empowerment and a forum to handle conflict amicably so that it does not interfere with our progress’* said one doctoral student.

**Table 4:** High priority areas for doctoral training workshops at Makerere University College of Health Sciences

**High priority areas**	**Mentors**	**Doctoral students**
**Procurement processes**	● Workshops with the procurement team to streamline the processes and encourage mutual benefit	● Finance management and budgeting modules
**National Drug Authority (NDA) regulatory processes**	● Meetings with NDA to review and design support systems to encourage timely review and feedback.	● Ongoing training in Human Subjects’ Research
	● Orientation on other research regulatory processes such as Institutional Review Boards (IRB) and the Uganda National Council for Science and Technology (UNCST).	
**Communication skills**	● Communication seminars	● Induction workshops for doctoral candidates and their mentors
	● Inspirational talks to doctoral students	
	● Inspirational talks to mentors	● Compile a frequently asked questions (FAQ) document on the doctoral students’ website
		● Training on conflict resolution and public relations

#### Limited human resource for health care delivery

**‘***The high load of clinical rounds and calls leaves me with little time for my doctoral training activities*’ said one doctoral student. ‘*However, there are few clinicians on my ward and patients need me’* the doctoral student added. The mentors suggested that clinical duties should be reduced for doctoral students to allow them protected time to pursue their training activities. One mentor said, ‘*The doctoral student does not have to do clinical duties and coordinate an entire course but he/she can participate in student tutorials, seminars and examinations’*.

## Discussion

### Peer support and collaboration with mentors improves retention and completion in doctoral programs

Both doctoral students and mentors identified with the increasing numbers of doctoral students and mentors. With increasing numbers, trainees reported the benefit of peer support through doctoral seminars to share challenges and innovative solutions. Our data is consistent with reports from a survey among 108 doctoral students in eight universities in the USA where half of all doctoral students were failing to complete their programs. In the latter survey, peer mentoring through cohorts of doctoral students and doctoral faculty was used to improve retention in doctoral programs [10]. In addition, there is evidence that networking and psychological support increased students’ productivity and satisfaction with their graduate school experience [11]. Similarly, in this study, both doctoral students and mentors advocated for regular inspirational and peer support activities.

### Skills’ training

Both doctoral students and mentors identified a gap in their communication skills and highlighted conflict resolution as a high priority area to be addressed in order to improve the doctoral training experience. Conflict may arise in cases of unmet expectations from the mentor/mentee, failure to meet timelines, unusual demands from mentees, mentees frustrations and absence of a systematic framework for resolving such conflict. Our findings mirror recent reports that disagreements between doctoral student-mentor pairs slow students’ progress and increase attrition rates among doctoral students [12]. Thus, good communication between the doctoral student and mentor would empower students to take major responsibility in decision-making and confidently navigate through the high demands and challenges within their respective disciplines [13]. In addition, it is important to note that close communication and collaboration between students and faculty improves task completion whilst it promotes team building practices [10]. We therefore recommend emphasis on communication training as well as interactive activities to promote networking among doctoral students and mentors.

Cross-cutting multidisciplinary courses including research regulatory compliance, budgeting and finance management should be encouraged to equip doctoral students with project management skills. Similarly, the doctoral students need knowledge and skills to handle the institutional procurement systems, another area that affects students’ progress [6]. A multi-disciplinary approach is clearly needed to mentor and prepare doctoral students for the multi-disciplinary challenges in global health leadership.

### Infrastructural development and human resource capacity

Infrastructure and human resource remains critical to the advancement of higher education in developing countries. Limited human resource capacity for health care delivery makes it challenging for clinicians to combine clinical duties with doctoral training. There is clearly need for training of more clinicians and researchers to meet the overwhelming demand for clinical care and subsequently increase the pool of mentors [4]. Sustainable research capacity building in developing countries requires multi-level training and supportive institutional infrastructure to empower African scientists to ask and answer regionally relevant research questions [2,4]. Investments to develop an academic environment are timely to increase scientists’ access to equipped research laboratories, internet connectivity, reference libraries, international journals, e-books, and access to world experts as mentors; among other needs [2]. Similarly, there is need for equivalent efforts to retain the trained professionals to mitigate the prevalent brain drain as locally trained clinicians, scientists and academicians migrate to work abroad in avoidance of the infrastructure and logistical challenges in developing countries [14-16].

### Limitations

This study was limited to data collected during the workshop that was attended by approximately a third of the doctoral students and their mentors. This sample was representative of the doctoral students and mentors at MakCHS since invitations were sent to the entire college email list. The 33% response rate from mentors is slightly higher than our previous response rate of 22% when a mentorship survey was conducted among mentors and mentees through the Uganda Fogarty Alumni [4]. The strength of the study was seeking opinions from both mentors and mentees in homogenous FGDs to allow discussion of a wide range of issues. We did not tape-record or video-record FGDs since it was not part of routine monitoring and evaluation of the doctoral training program. However all participants’ responses were recorded by the note-takers verbatim. In addition, recorded data was presented in the subsequent team discussion to allow additions and clarifications.

## Conclusion

Systemic and infrastructural limitations affect the quality of the doctoral training experience at MaKCHS. Clinical and biomedical research infrastructure, in addition to training in research regulatory processes, procurement and finance management, communication skills and information technology were highlighted as high priority areas for strategic interventions to improve mentoring within doctoral training programs.

## Competing interests

The authors do not report any competing interests.

## Authors’ contribution

DN, AK, DKK, EO, MK, HMK and NS conceptualised the idea, DN, AK, EO, DKK and HMK contributed to the data analysis and interpretation. DN drafted the manuscript. All authors reviewed the manuscript and approved the final version for publication.

## Pre-publication history

The pre-publication history for this paper can be accessed here:

http://www.biomedcentral.com/1472-6920/14/9/prepub
